# Developmental changes in the transcriptome of the rat choroid plexus in relation to neuroprotection

**DOI:** 10.1186/2045-8118-10-25

**Published:** 2013-08-01

**Authors:** Ingrid Kratzer, Shane A Liddelow, Norman R Saunders, Kate M Dziegielewska, Nathalie Strazielle, Jean-Francois Ghersi-Egea

**Affiliations:** 1 Inserm U1028, Lyon Neuroscience Research Center, Neurooncology & Neuroinflammation Team, Lyon-1 University, Lyon F-69000, France; 2 Department of Pharmacology & Therapeutics, University of Melbourne, Parkville, Victoria 3010, Australia; 3 Department of Neurobiology, Stanford University, Stanford, CA 94305, USA; 4 Brain-i, Lyon, France

**Keywords:** Cerebrospinal fluid, Development, Efflux transporter, Multidrug resistance, Detoxification, Drug metabolizing enzymes, Antioxidant

## Abstract

**Background:**

The choroid plexuses are the interface between the blood and the cerebrospinal fluid (CSF) contained within the ventricular spaces of the central nervous system. The tight junctions linking adjacent cells of the choroidal epithelium create a physical barrier to paracellular movement of molecules. Multispecific efflux transporters as well as drug-metabolizing and antioxidant enzymes functioning in these cells contribute to a metabolic barrier. These barrier properties reflect a neuroprotective function of the choroid plexus. The choroid plexuses develop early during embryogenesis and provide pivotal control of the internal environment throughout development when the brain is especially vulnerable to toxic insults. Perinatal injuries like hypoxia and trauma, and exposure to drugs or toxic xenobiotics can have serious consequences on neurogenesis and long-term development. The present study describes the developmental expression pattern of genes involved in the neuroprotective functions of the blood–CSF barrier.

**Methods:**

The transcriptome of rat lateral ventricular choroid plexuses isolated from fifteen-day-old embryos, nineteen-day old fetuses, two-day old pups, and adults was analyzed by a combination of Affymetrix microarrays, Illumina RNA-Sequencing, and quantitative RT-PCR.

**Results:**

Genes coding for proteins involved in junction formation are expressed early during development. Overall perinatal expression levels of genes involved in drug metabolism and antioxidant mechanisms are similar to, or higher than levels measured in adults. A similar developmental pattern was observed for multispecific efflux transporter genes of the *Abc* and *Slc* superfamilies. Expression of all these genes was more variable in choroid plexus from fifteen-day-old embryos. A large panel of transcription factors involved in the xenobiotic- or cell stress-mediated induction of detoxifying enzymes and transporters is also expressed throughout development.

**Conclusions:**

This transcriptomic analysis suggests relatively well–established neuroprotective mechanisms at the blood-CSF barrier throughout development of the rat. The expression of many transcription factors early in development raises the possibility of additional protection for the vulnerable developing brain, should the fetus or newborn be exposed to drugs or other xenobiotics.

## Background

Choroid plexuses form the main interface between the blood and cerebrospinal fluid (CSF) and participate in the control of brain homeostasis. In the rat, choroid plexuses of the fourth, lateral, and third ventricles appear in embryos on day 12, 13 and 16, respectively [1]. In human, choroid plexuses develop around week 6–7 of gestation [2]. Selective influx transport and secretion mechanisms confer on the choroidal epithelium an important role in supply of nutrient and bioactive molecules to the developing brain [3,4]. The choroid plexuses also produce and secrete most of the CSF through the combined activity of various enzymes, transporters and ion channels located in the epithelial cells [5]. The rate of CSF secretion increases around birth in most mammals [6,7].

**Figure 1:**
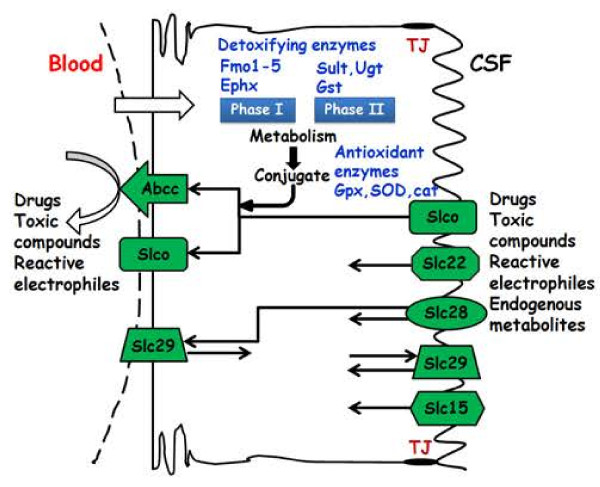
**Schematic representation of neuroprotective mechanisms at the blood-CSF barrier.** In addition to tight junctions (red) that prevent non-specific paracellular diffusion, a wide variety of multispecific efflux transporters (green), as well as antioxidant and drug metabolizing enzymes (blue), participate in the neuroprotective and detoxifying functions of the choroid plexus. Only the most documented mechanisms are represented on the scheme (see text for details and Table 1 for a description of enzyme and transporter families).

In addition to these secreting and supplying activities, the choroid plexuses fulfill neuroprotective functions both as a physical and as a biochemical barrier between the blood and the nascent CSF [8] (Figure 1). Transmission electron microscopy combined with the use of electron–dense tracers [9] and freeze–fracture electron microscopy [10,11] demonstrated that the tight junctions (TJ), which link adjacent epithelial cells, constitute the anatomical basis of this barrier. This physical barrier appears efficient in preventing the paracellular leakage of low molecular weight molecules from the earliest stages of plexus development [12-14]. The molecular composition of choroidal TJs is regulated during development, as exemplified by the coordinated up– or down–regulation of specific pore–forming and tightening claudins that make up the core of the junctional complex [15]. In addition to this physical barrier, a number of antioxidant and drug metabolizing enzymes, and a wide variety of multispecific efflux transporters participate in the neuroprotective and detoxifying functions (Figure 1, Table 1). Efflux transporters eliminate endogenous bioactive metabolites and xenobiotics from the CSF or prevent their entry through the choroidal epithelium. They include primary energy-dependent unidirectional transport pumps of the ATP-binding cassette (ABC) transporter superfamily such as Abcc1 ^a^ and Abcc4 [16,17], as well as multispecific transporters of the solute carrier (Slc) family, such as Slco1a5, Slc22a8 [18,19], or Slc15a2 [20]. Activities of the antioxidant enzymes epoxide hydrolases and glutathione peroxidases, and of drug-conjugating enzymes are high in the choroid plexuses [21]. Conjugation to glutathione or glucuronic acid, coupled with a basolateral efflux of the produced metabolites, forms an efficient enzymatic barrier to the entry of conjugating enzyme substrates into the CSF [22,23].

**Table 1:** List of gene official symbols and aliases

***Gene-symbol***	**Aliases**	**mRNA ID**
**ABC transporters**		
*Abcb1b*	Mdr1, Pgy1, Abcb1	NM_012623
*Abcc1*	Mrp1	NM_022281
*Abcc3*	Mlp2, Mrp3	NM_080581
*Abcc4*	Mrp4	NM_133411
*Abcc5*	Mrp5, Abcc5a	NM_053924
*Abcc9*	Sur2	NM_013040
*Abcc10*	Mrp7	NM_001108201
*Abcg2*	BCRP1	NM_181381
**Solute carriers: organic anion transporting polypeptides**		
*Slco2a1*	Slc21a2, Matr1	NM_022667
*Slco1a3*	Slc21a4	NM_030837
*Slco1a4*	Slc21a5, Oatp2	NM_131906
*Slco1a5*	Slc21a7, Oatp3	NM_030838
*Slco2b1*	Slc21a9, moat1	NM_080786
*Slco3a1*	Slc21a11, Oatp3a1	NM_177481
*Slco1c1*	Bsat1, Slc21a14, Oatp14	NM_053441
**Solute carriers: organic anion /cation/zwitterion transporters**		
*Slc22a2*	OCT2	NM_031584
*Slc22a4*	Octn1	NM_022270
*Slc22a5*	Octn2, CT1, UST2r	NM_019269
*Slc22a6*	Oat1, Paht, Roat, Orctl1	NM_017224
*Slc22a7*	Oat2	NM_053537
*Slc22a8*	Oat3, Roct	NM_031332
*Slc22a12*	Rst	NM_001034943
*Slc22a15*		NM_001107707
*Slc22a17*	Boct, 24p3R	NM_177421
*Slc22a25*	Ust1, Slc22a9	NM_138908
**Solute carriers: nucleoside and peptide transporters**		
*Slc15a2*	Pept2	NM_031672
*Slc28a2*	Cnt2	NM_031664
*Slc28a3*	Cnt3	NM_080908
*Slc29a1*	Ent1	NM_031684
*Slc29a2*	Ent2	NM_031738
*Slc29a3*	Ent3	NM_181639
*Slc29a4*		NM_001105911
**Cytochrome P450s, epoxide hydrolases, flavin-containing monooxygenases**		
*Cyp1b1*		NM_012940
*Cyp2d4*	Cyp2d6, Cyp2d18 Cyp2d22	NM_138515
*Cyp2j4*	CYP2J2	NM_023025
*Cyp2u1*		NM_001024779
*Ephx1*, transcript variant 1	MEH8	NM_001034090
*Ephx1*, transcript variant 2		NM_012844
*Ephx2*	CEH, SEH	NM_022936
*Fmo1*	RFMO1A	NM_012792
*Fmo2*		NM_144737
*Fmo3*		NM_053433
*Fmo4*, transcript variant 1		NM_144562
*Fmo4*, transcript variant 2		NM_144561
*Fmo5*		NM_144739
**UDP-glucuronosyltransferases and sulfotransferases transferases**		
*Ugt1a1*	UDPGT 1-1	NM_012683
*Ugt1a2*	UDPGT 1-2	NM_201423
*Ugt1a3*	UDPGT 1-3	NM_201424
*Ugt1a5*	UDPGT 1-5	NM_001039549
*Ugt1a6*	UDPGT 1-6	NM_057105
*Ugt1a7c*	UDPGT 1-7	NM_130407
*Ugt1a8*	UDPGT 1-8	NM_175846
*Ugt1a9*		NM_201425
*Ugt2a1*	Ugt2a1p	NM_022228
*Sult1a1*	Stm, Stp1, ASTIV, Mx-ST, PST-1	NM_031834
*Sut1d1*	Sultn, Sult-n	NM_021769
*Sult5a1*		NM_001106194
**Glutathione S-transferases (GSTs) and glutathione-synthesis related genes**		
*Gsta1*		NM_017013
*Gsta2*		LOC494499
*Gsta3*	Yc1	NM_031509
*Gsta4*		NM_001106840
*Gstm1*		NM_017014
*Gstm2*		NM_177426
*Gstm3*	GstYb4	NM_020540
*Gstm4*		NM_001024304
*Gstm5*		NM_172038
*Gstm7*		NM_031154
*Gstp1*	Gst3, Gstp, Gstp2	NM_012577
*Mgst1*		NM_134349
*Mgst2*		NM_001106430
*Mgst3*		NM_001191594
*Gss*		NM_012962
*Gclc*	Glclc	NM_012815
*Gclm*	Glclr	NM_017305
**Heme oxygenases, biliverdin reductase**		
*Hmox1*	Ho1, Heox, Hmox, Ho-1, HEOXG	NM_012580
*Hmox2*	Ho-2	NM_024387
*Hmox3*	HO-3	AF058787
*Blvra*		NM_053850
*Blvrb*		NM_001106236
**Glutathione peroxidases and –reductase**		
*Gpx1*	GSHPx, GSHPx-1	NM_030826
*Gpx3*	Gpxp, GPx-P, GSHPx-3, GSHPx-P	NM_022525
*Gpx4*	Phgpx, gpx-4, snGpx	NM_017165
*Gpx7*		NM_001106673
*Gpx8*		NM_001106411
*Gsr*		NM_053906
*Sod1*	CuZnSOD	NM_017050
*Sod2*		NM_017051
*Sod3*		NM_012880
*Cat*	CS1, Cas1,Catl, Cs-1, Cat01	NM_012520
**Regulatory transcription factors**		
*Nr1i2*	PXR	NM_052980
*Nr1i3*	CAR	NM_022941
*Rxra*		NM_012805
*Ppara*	Nr1c1, Ppar	NM_013196
*Nr3c1*	GR, Grc, Grl	NM_012576
*Cebpb*	LAP, TCF5, Il6dbp, NF-IL6	NM_024125
*Ahr*	AhR	NM_013149
*Arnt*	Arnt1	NM_012780
*Nfe2l2*	Nrf2	NM_031789
*Hif1a*	MOP1	NM_024359
*Tp53*	p53, Trp53	NM_030989
**Monoamine oxidases, dopa decarboxylase, catechol-O-methyltransferase**		
*Maoa*	Mao	NM_033653
*Maob*		NM_013198
*Ddc*		NM_012545
*Comt*		NM_012531
**Tight junction-associated genes**		
*Cldn1*		NM_031699
*Cldn2*		NM_001106846
*Cldn3*		NM_031700
*Cldn6*		NM_001102364
*Cldn9*		NM_001011889
*Cldn10*		NM_001106058
*Cldn11*		NM_053457
*Cldn12*		NM_001100813
*Cldn19*		NM_001008514
*Cldn22*		NM_001110143
*Marveld1*	Mrvldc1	NM_001107590
*Marveld2*	Mrvldc2, Tric	NM_001108936
*Marveld3*		NM_001109132
*Ocln*		NM_031329
*Lsr*	Lisch7	NM_032616
*Tjp1*	ZO-1	NM_001106266
*Tjp2*	ZO-2	NM_053773
*Tjp3*	ZO-3	NM_001108073
*F11r*	Jam1	NM_053796
*Jam2*		NM_001034004

The molecular identification of all transporters and drug metabolizing enzymes in the choroid plexus fulfilling a biochemical barrier function at the interface between blood and CSF remains incomplete. Data on the developmental expression pattern of these genes are even scarcer. *Abcc1*, *Abcc4* and *Abcg2* transcripts are detected in rat choroid plexuses as early as embryonic day 15. *Abcg2* expression is highest at this stage and subsequently declines in the adult [24]. The *Abcc1* gene is well expressed and its protein product already localized at the basolateral blood–facing membrane of the choroidal epithelium in both newborn rats [24,25], and human neonates [26]. The enzymatic activity of the sulfotransferase Sult1a1, which conjugates phenolic drugs with sulfate, is high in human fetal choroid plexuses [27]. High glutathione-*S*-transferase (GST) activity is detected in choroid plexuses of newborn rats and human fetuses [23]. These currently available data on choroidal transport and detoxification activities at embryonic and perinatal stages suggest that a functional biochemical barrier supported by various enzymatic pathways could contribute substantially to the protection of the developing brain.

The central nervous system is especially vulnerable to perinatal injuries, including hypoxia, systemic inflammation and traumatic brain injury. Maternal exposure to neurotoxic compounds and drugs can have dramatic consequences for neurogenesis, leading to irreparable long-term neurodevelopmental disorders [28]. The purpose of the present study was to expand our knowledge of neuroprotective mechanisms at the blood-CSF barrier in the developing brain and our understanding of the factors setting the cerebral bioavailability of drugs in the context of pediatric treatments. We have established the developmental expression profiles of genes involved in drug transport and detoxification in the choroidal tissue. We have combined data obtained from Illumina RNA-Seq (high throughput RNA sequencing) and Affymetrix microarray technologies developed in two independent laboratories and analyzed by quantitative real-time PCR (qRT-PCR) additional transcripts not represented on the array. The genes included in the analysis were selected for their involvement in drug/xenobiotic transport or in detoxification pathways. The transcription of some of these genes is known to be regulated in peripheral organs under various physiological and pathological stimuli, and can also be pharmacologically modulated [29-31]. For this reason, transcription factors involved in the regulation of drug metabolizing enzymes and transporters were included in this study.

## Materials and methods

### Tissue collection and RNA extraction

Animal care and procedures were conducted according to the guidelines approved by the French ethical committee (decree 87–848), by the European Community (directive 86-609-EEC) and the University of Melbourne Animal Ethics Committee based on National Health and Medical Research Council guidelines. Sprague–Dawley rats, either adult males, pregnant time-dated females, or females with their litter, were obtained from Janvier (Le Genest Saint Isle, France) or the Biomedical Research Facility at the University of Melbourne (Victoria, Australia). All animals were kept under similar conditions in standard cages, with free access to food and tap water under a controlled environment (12 h day/light cycles). Choroid plexuses of the lateral ventricle were dissected under a stereomicroscope from two-day-old (P2) and adult rats as previously described and illustrated [3,22,32]. Timed-pregnant female rats were anesthetized with inhaled isoflurane and body temperature maintained with a heated pad. Fifteen-day-old (E15) embryos and nineteen-day-old (E19) fetuses were removed one by one from the mother and used for brain sampling and microdissection of the choroid plexuses [33]. All steps were performed under RNAse-free conditions. The collected tissues were snap-frozen in liquid nitrogen and kept at −80°C until use.

For Affymetrix microarrays, choroid plexuses were pooled from 3 (adult) or 5 (developing) animals. Total RNA was isolated from two pools of choroid plexuses sampled from E19, P2, or adult rats using the RNeasy® Micro Kit (Qiagen, Valencia, CA, USA), and DNAse-treated according to the manufacturer’s protocol. qRT-PCR analysis was performed on four (P2, adult) or three (embryonic) pools of mRNA. For Illumina RNA-Seq, three pooled samples of ten lateral ventricular choroid plexuses from each E15 and adult age were used. Total RNA was extracted using the RNeasy Plus Mini Kit, Qiashredder columns and gDNA removal columns (Qiagen, Valencia, CA, USA) according to standard supplier protocols. All RNA samples were quantified using a NanoDrop 2000c spectrophotometer (Thermo Scientific, Wilmington, DE, USA) and quality checked with the Agilent 2100 Bioanalyser (Agilent Technologies, Palo Alto, CA, USA).

### Affymetrix microarray

Microarray analysis was performed using a high-density oligonucleotide array (GeneChip Rat Genome 230 2.0 array, Affymetrix, Santa Clara, CA, USA). Total RNA (100 ng) was amplified and biotin-labeled using GeneChip® 3’ IVT Express target labeling and control reagents according to Affymetrix protocol (http://www.affymetrix.com). Before amplification, all samples were spiked with synthetic mRNAs at different concentrations, which were used as positive controls to ascertain the quality of the process. Biotinylated antisense cRNA for microarray hybridization was prepared. After final purification using magnetic beads, cRNAs were quantified using a NanoDrop and quality checked with Agilent 2100 Bioanalyzer. Hybridization was performed according to the Affymetrix protocol. Briefly, 10 μg of labelled cRNA was fragmented and denaturated in hybridization buffer, then hybridized on the chip for 6 h at 45°C with constant mixing by rotation at 60 rpm in an Genechip hybridization oven 640 (Affymetrix). After hybridization, arrays were washed and stained with streptavidin-phycoerythrin (GeneChip® Hybridization Wash and Stain Kit) in a fluidic 450 (Affymetrix) according to the manufacturer’s instruction. The arrays were read with a confocal laser (Genechip scanner 3000, Affymetrix). CEL files summarizing the probe cell intensity data were generated using the Affymetrix GeneChip Command Console software 3.0. Data were normalized with Affymetrix Expression Console software using MAS5 statistical algorithm. Data have been deposited into the Gene Expression Omnibus repository (http://www.ncbi.nlm.nih.gov/geo) under accession number GSE44056.

### Illumina RNA-Seq

RNA sequencing was performed at the Australian Genome Research Facility (Melbourne, VIC, Australia). A cDNA library was prepared from 10 μg of total RNA using the mRNA-Seq Sample Preparation Kit (Illumina, San Diego, CA, USA) according to standard manufacturer protocol. Quality of the library was verified using a DNA 1000 chip using the Agilent 2100 Bioanalyzer (Agilent) and quantified by fluorimetry. Illumina technology allows both identification of the mRNAs present in the total RNA preparations analyzed, and provides a relative number of mRNA copies for these genes in each of the different preparations. In brief, the library was subjected to 100 bp single end read cycles of sequencing on an Illumina Genome Analyzer IIx (Illumina) as per manufacturer protocol. Cluster generation was performed on a c-Bot (Illumina) with a single read cluster generation kit. Refer to [34] for full methods and bioanalysis. Details of the analysis of the output from the high throughput RNA-Seq are published separately [34]. Briefly, reads were trimmed to remove ambiguous bases from the start and segments with low quality scores from the end. Trimmed reads were mapped with Bowtie version 0.12.7 to the Ensembl rat genome, release 61, and reads that did not map uniquely were discarded. The number of reads mapped to nuclear genes was determined with HTSeq version 0.4.7p4, using the default “union” counting option. Data have been deposited into the Gene Expression Omnibus repository under accession code GSE44072.

### Quantitative real-time PCR

RNA (1 μg) was spiked with 25 pg of bacterial AraB RNA from *E.coli* (GE Healthcare Bio-Sciences Freiburg, Germany), used as an external standard for normalization, as the expression of conventionally used house-keeping genes proved to be variable between developmental stages. RNA was reverse transcribed using the iScript Reverse Transcription Supermix (Bio-Rad, Hercules, CA, USA). Protocols for qRT-PCR performed with the LightCycler FastStart-DNA Master SYBR Green I kit in the LightCycler® 1.5 Instrument (Roche Diagnostics GmbH, Mannheim, Germany), data analysis, and statistics have been described previously [15]. The number of biological replicates used was sufficient to assess a statistical significance for a Log_2_FC_ad_ value of 1 with a statistical power averaging 95% (alpha error level set at 0.05) for all genes whose level of expression was defined by a crossing-point lower than 32. All primers were designed using NCBI Primer-BLAST and selected to generate amplicons with a length of 100 to 200 bp. A list of primers, amplicon sizes and MgCl_2_-concentrations used for qRT-PCR is given in Additional file 1.

### Data presentation

Adult choroid plexuses were used as reference sample for all three techniques. On all graphs, data are expressed as Log_2_-transformed fold changes (FC) of expression levels in developing versus adult choroid plexus (Log_2_ FC_ad_). Negative and positive values indicate lower and higher expression in choroid plexuses of developing animals than in choroid plexuses of adult rats, respectively. Data are means of two (Affymetrix), three (Illumina) or more (qRT-PCR) values, obtained from different RNA preparations.

Genes detected with a fluorescent intensity value in adult choroid plexus higher than 1000 on the Affymetrix arrays were considered highly expressed and are indicated in bold in the figures. Genes with a value between 100 and 200, i.e. close to background (mean value of 60), or with a crossing point value above 30 by qRT-PCR, were considered to be expressed at low level and are indicated in parentheses. As a reference, the *transthyretin* gene, which product is one of the most abundant proteins synthesized by choroid plexus, was the gene with the 100^th^ highest level of expression in adult on the array, with a fluorescent intensity value of 13244, and had a crossing point of 12 in our amplification conditions. The classification is only indicative, as the hybridization efficacy may vary from one probe to another.

## Results and discussion

### Combined analysis of gene expression data by Affymetrix microarray, Illumina RNA-sequencing, and qRT-PCR

The expression level of selected genes was assessed in E19, P2, and adult rat lateral ventricular choroid plexus using Affymetrix microarrays, and in E15 and adult lateral ventricular choroid plexus by Illumina RNA-Seq analysis. Combining the two techniques enabled the inclusion of a larger number of genes in the study as some of these genes were identified by either Affymetrix or Illumina technology only. Some choroidal transcripts of special relevance to possible brain protection, but not represented on the arrays, were analyzed by qRT-PCR. For each gene, the adult stage included in all methods of analysis served as a reference to normalize the data set. Individual developmental profiles were built from the Log_2_ FC_ad_ mean values calculated at each stage. The profiles are grouped and interpreted for families of genes with similar functions.

To optimize the reliability of the data generated by microarrays, mRNA samples were prepared from two separate pools of choroid plexuses, each being collected from several animals. The choroidal tissue was carefully controlled with a stereomicroscope to verify the absence of contaminating tissue. Performance of the amplification/labeling reaction and of hybridization was assessed by external spike-in controls (exogenous RNAs added to the mRNAs samples before amplification, and biotin-labeled cRNA added prior to hybridization). The perfect alignment of signals detected for these spikes along the line of identity on plots comparing duplicate microarrays validates the technical reproducibility of the data (not shown). The duplicate choroidal mRNA populations analyzed for each stage were highly similar as the expression level of 99.4% of all genes detected at level above a background of 100 fluorescence units as defined by Affymetrix software differed by less than a factor of two between samples (not shown). Finally, the expression levels of the neuroprotective genes reported in this study differed only by 12.4 ± 1.4% (mean ± SEM) between the two matching samples.

The qRT-PCR and Illumina RNA-Seq methods have some advantages over the microarray technology. Being both based on the relative quantification of the number of mRNA copies, they generate similar fold-changes [35,36]. They offer a larger dynamic and linear range of expression levels and are in theory more accurate for quantifying gene expression. Owing to the absence of standard curves compared with qRT-PCR, and the higher background compared to RNA sequencing, the microarray technology might yield fold changes with lower accuracy. We further tested the reliability of our Affymetrix microarray data by comparing the E19-to-adult fold changes obtained by this technique to those calculated by qRT-PCR (*n* = 3 for E19, n = 4 for other stages) for eighteen genes related to intercellular junctions, transport and detoxification (*Cldn1, Cldn3, Cldn5, Cldn11, Cldn12, Cldn19, Ocln, Tjp1, Tjp2, Tjp3, Abcc4, Abcg2, Slco1a4, Slco1a5, Slco1c1, Slc22a17, Slc22a8, Ephx1*). For all genes, both Log_2_ FC_ad_ values were of the same sign, either positive or negative. Furthermore, the average variation between the two Log_2_ FC_ad_ values calculated for the 18 genes was low (31.7 ± 9%, mean ± SEM). In the figures, only microarray data are presented for these genes.

Altogether, these results provided a good indication that Affymetrix data could be reliably combined with qRT-PCR and Illumina RNA sequencing data, therefore enabling a most comprehensive gene expression analysis by reducing the number of false-negative genes associated with each of the techniques. Previous similar studies comparing Affymetrix microarrays and Illumina RNA-Seq to analyze gene expression showed that the two methods generate reasonably similar data, with RNA sequencing identifying additional genes not represented in the microarrays [37,38].

### Developmental profile of TJ-associated genes

**Figure 2:**
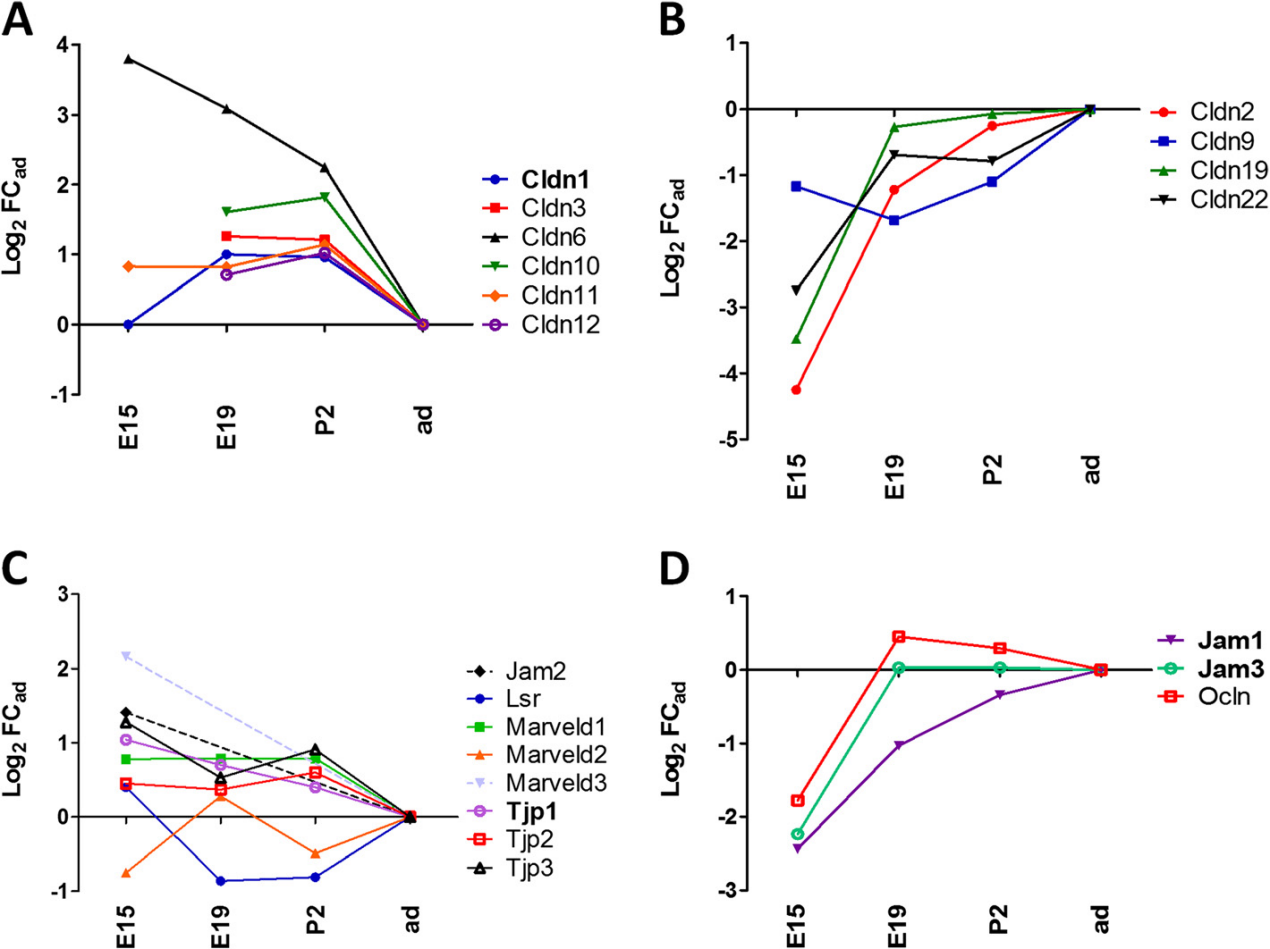
**Developmental profiles of tight junction-associated genes in rat choroid plexus.** Analysis was performed on lateral ventricle choroid plexus at four developmental stages. Affymetrix and Illumina RNA-Seq techniques were combined and data expressed as Log_2_ values of fold change relative to adult (Log_2_ FC_ad_). For selective genes that were absent in Affymetrix chips, qRT-PCR was performed. Strongly expressed genes are indicated in bold. **(A** and **B)** show gene expression profiles for claudins with decreasing and increasing expression during development, respectively. **(C** and **D)** show gene expression profiles for selected other TJ-associated protein transcripts. Abbreviations: *E15,* embryonic day 15; *E19,* embryonic day 19; P2, postnatal day 2; *ad,* adult. Correspondences between gene symbols, common names, and accession numbers are listed in Table 1.

The restriction of intercellular diffusion is a mandatory prerequisite for effective and regulated transport across cellular barriers. At both early embryonic (E15) and perinatal (E19 and P2) stages, a large number of TJ-associated genes were expressed in choroid plexuses at a level equal or higher than that measured at the adult stage (Figure 2A and C). These genes included the highly expressed *cldn1* and *cldn3.* The expression of seven other genes was lower in E15 rats than in the adult, in particular that of *cldn2* (Figure 2B and D). Of note, for most of these TJ-associated genes, the FC absolute values were largely smaller at E19 than E15, reflecting the establishment of a mature, adult-like phenotype before birth. These results are congruent with our previous data that showed an early formation of complex TJs between choroidal epithelial cells, and a remodeling of the TJ protein composition around birth in rat [12,15]. An in-depth analysis of the genes involved in the formation, maintenance and regulation of choroidal tight junctions during early brain development is reported in another article [34].

### Developmental profile of efflux transporters

In this analysis we define transporters involved in brain protection as efflux transporters that accept drugs and other xenobiotics for substrates and display a broad specificity. Within ABC transporters, the *Abcb1a/b*, *Abcg2*, and *Abcc* genes meet these criteria, as they are responsible for the efflux of numerous endo- and xenobiotics and of glutathione-, glucurono-, and sulfo-conjugates [8,39]. Several Slc subfamilies, which also limit the cerebral availability of numerous compounds, were selected in the study. Slco transporters carry amphiphilic anionic drugs and various glucurono-, sulfo-, and glutathione-conjugates. Slc22 proteins transport a large range of both small relatively hydrophilic organic anions and organic cations including β-lactam antiobiotics, non-steroidal anti-inflammatory drugs, and antiviral nucleoside reverse transcriptase inhibitors [18,19]. Dipeptide transporters of the Slc15 subfamily and nucleoside transporters belonging to both Slc28 and Slc29 subfamilies were also incorporated in the study, as they transport a number of antiviral and/or nucleoside-derived drugs in addition to their typical endogenous substrates [18,40].

#### ABC transporters

Several multispecific efflux ABC transporters have been located at the blood–brain interfaces where they limit the entry of harmful toxins, but also of pharmacologic agents such as anticancer drugs or antiretroviral protease inhibitors [16,17,39].

**Figure 3:**
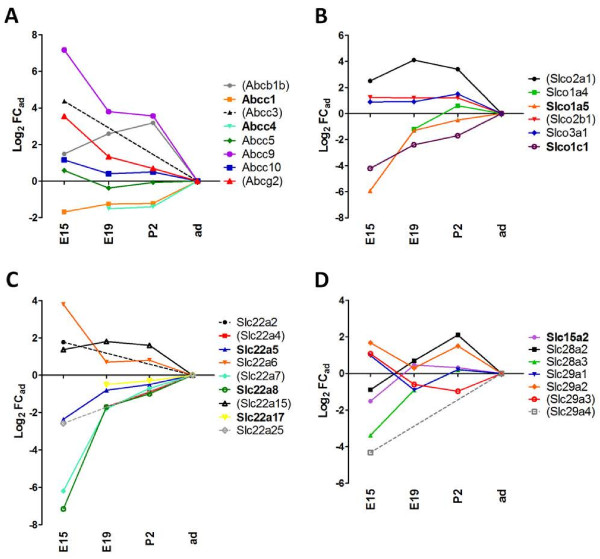
**Developmental profiles of Abc and Slc efflux transporters. (A) shows gene expression profiles for *Abc *genes.***Abcc3* was expressed at E19 and at P2, but was not detected in adult by Affymetrix analysis. **(B)** shows gene expression profiles for the *Slco* genes. **(C)** shows gene expression profiles for the *Slc22* genes. *Slc22a4* displayed the same Log_2_ FC_ad_ as S*lc22a7* at E19, P2 and adult, thus profiles are overlapping. *Slc22a12* was detected at E15, but not in adult by Illumina RNA-Seq method. **(D)** shows gene expression profiles for *Slc15*, *Slc28,* and *Slc29* genes. Strongly expressed genes are indicated in bold. Genes indicated in parentheses have an apparent very low expression level in adult choroid plexus. Abbreviations, data analysis, and data expression as in Figure 2.

*Abcc1* and *Abcc4* transcripts were abundant in choroid plexuses throughout development, and were moderately enriched in the adult compared to earlier stages. In contrast, the four other *Abcc* genes were expressed at a similar or higher level during development compared to adult. In particular, *Abcc9* transcripts were strikingly enriched in earlier stages, with a 144-fold higher level in E15 compared to adult (p < 0.001). Expression levels of *Abcb1b* and *Abcg2* were also higher at E15 and perinatal stages (qRT-PCR: p < 0.05) than in adult, in which they were apparently very low (Figure 3A).

These results indicate that the main Abc transporter genes expressed in choroid plexus belong to the *Abcc* subfamily. This is in accordance with a previous report showing the expression of *Abcc1, Abcc4, Abcc5,* and to a lesser extent *Abcc3* in this tissue in adult rat [41]. It is also in agreement with the expression of these genes in both embryonic and adult mouse choroid plexuses [4]. Abcc1 protein was largely enriched in mouse, rat and human choroid plexuses compared to other brain tissues, as shown by immunohistochemistry and Western Blot [16,25]. *In vivo* transport experiments using knockout mice pointed to the role of this carrier in limiting the CSF concentration of drugs such as etoposide in the CSF [16]. In mouse, Abcc4 protein was located in both the luminal membrane of brain capillaries and the basolateral membrane of the choroidal epithelium [17]. Microdialysis studies showed that this carrier strongly restricts the passage of drugs such as topotecan from the blood into the CSF [17]. The developmental patterns observed also corroborate previous data showing substantial expression levels of *Abcc1* and *Abcc4* in newborn rat choroid plexus [23], and the developmental up-regulation observed between E15 and adult for both *Abcc* genes [24]. Despite the developmental regulation of *Abcc1* level of expression, Abcc1 protein level was already as high in P2 rats as in adult and was also located at the basolateral membrane of the choroid plexus [25]. Overall, the Abcc developmental profiles suggest that in addition to the main Abcc1, Abcc4 and other Abcc transporters may have a crucial function as efflux pumps during early brain development.

The low level of *Abcb1* transcripts in adult rat choroid plexus is in line with earlier findings that showed, using Western blot analysis of adult rat and human tissues, that Abcb1 protein was far less abundant in choroid plexus by comparison to microvessels [25]. Similarly, the low level of expression of *Abcg2* that we report in adult choroid plexus is accordant with the differential immunoreactivity of microvessels (highly positive) compared to choroid plexuses (negative), observed for this transporter in adult rat brain [24,42]. The higher expression of both *Abcb1* and *Abcg2* from early embryonic to postnatal stages (Figure 3A) suggests that these transporters fulfill age-related functions at the choroid plexus. A similar enrichment of *Abcg2* transcripts in E15 compared to adult was described in mouse choroid plexuses [4], and Abcg2 immunoreactivity was detected in embryonic rat choroid plexus, but not in the adult tissue [24,42]. The localization of the transporter at the basolateral membrane of the choroidal epithelial cells indicates that it could act as an efflux pump, similarly to Abcc1 and Abcc4. The precise localization of Abcb1 and other Abcc transporters identified in embryonic choroid plexus remains to be determined in order to understand the polarity of transport achieved by these proteins at the blood-CSF barrier.

#### Slco transporters

Members of the Slco subfamily, referred to as organic anion transporting polypeptides, mediate sodium-independent transport of relatively large amphipathic substrates such as taurocholate, thyroid hormones, leukotrienes, and various drugs among which non-steroidal anti-inflammatory drugs, the synthetic opioid peptide DPDPE, digoxin, or dexamethasone. They also transport conjugates of steroids, drugs, and other xenobiotics [43].

Within this family, *Slco1a5* and *Slco1c1* were highly expressed in rat choroid plexus (Figure 3B). They were both enriched in the adult compared to prenatal stages (RNA-Seq or qRT-PCR: p < 0.01). The expression level of *Slco1a5*, low at E15, rapidly increased to approach adult level by E19. *Slco1c1* transcript level increased more steadily between E15 and adult. Four additional genes of this family, *Slco1a4, Slco3a1, Slco2a1, and Slco2b1* were identified in choroid plexus, and expressed at equal or higher level in developing animals compared to adults.

Slco1a5 is a highly expressed choroidal transporter [18,41], which is located at the apical membrane of the choroid plexus epithelium [18,41]. It appears to play a major role in CSF-to-blood transport of organic anions [44]. The already substantial expression of *Slco1a5* at E19 compared to E15 suggests that the functional activity of this transporter is critical during the perinatal period. Slco1a4 protein has been previously detected in adult choroid plexus [45] where it is located at the basolateral membrane of choroidal epithelial cells [46]. Considering the partially overlapping substrate specificities of Slco1a5 and Slco1a4 [47], the polarized and opposite distribution of these transporters at both membrane domains of choroid plexus epithelial cells could be important for vectorial transport of ligands out of the CSF. Alternatively, the apical Slco1a5 protein may work in concert with basolateral Abcc transporters to achieve efficient CSF-to-blood efflux of organic anions.

Of note is the unexpected developmental regulation of *Slco1c1* at the choroid plexus. This carrier is a high affinity transporter of thyroxine, a thyroid hormone important for multiple neurodevelopmental processes. Thyroid hormone deficiency in the brain during the fetal and neonatal period can cause mental retardation [48,49]. The low expression of choroidal *Slco1c1* in the embryonic and perinatal stages suggests that other carriers are active at the blood-CSF barrier to provide for cerebral thyroxine requirements during early brain development [50]. *Slco3a1,* whose expression is slightly enriched in E19 and P2 compared to adult, and *Slc16a10*, expressed in fetal and newborn choroid plexuses, are potential candidates. Two different splice variants of the SLCO3A1 protein have been located at the apical and basolateral membranes of the human choroidal epithelium, and they both mediate thyroxine uptake in transfected cells and injected oocytes [51]. Slc16a10 is also an influx transporter for T3 and T4. It was recently identified in mouse embryo choroid plexus at a substantially (67-fold) higher level than in the adult [4]. Another likely candidate is transthyretin, whose synthesis in the brain is restricted to choroid plexus. The *transthyretin* gene is highly expressed throughout development in rat choroid plexus ([52] and data not shown), and this thyroxin carrier was shown to be important and necessary for various aspects of normal brain development [53].

#### Slc22 transporters

Members of the Slc22 subfamily are classified as organic anion transporters, organic cation transporters, and organic cation/carnitine transporters. Organic anion transporters are anion exchangers, and accept various toxins and drugs as substrates, i.e. antineoplastics, antihypertensives, non-steroidal anti-inflammatory drugs, and ß-lactam antibiotics, such as benzylpenicillin [54]. Organic cation transporters function as membrane potential-dependent uniporters that mediate the facilitated diffusion of endogenous amines such as dopamine, or therapeutic drugs such as morphine and antihistamines. Organic cation/carnitine transporters carry carnitine with variable affinities. Two members of this subfamily, octn1 and octn2, also accept different cationic xenobiotics including drugs as substrates [55].

*Slc22a5*, *Slc22a8*, and *Slc22a17* were highly expressed in rat choroid plexus from E15 onwards. Seven additional organic cation and anion transporter genes, *Slc22a2, Slc22a4, Slc22a6, Slc22a7, Slc22a12, Slc22a15,* and *Slc22a25* were found expressed in the choroidal tissue (Figure 3C). Transcripts were enriched in early embryos for four of these genes, and enriched in adult for four others. It should be noted that at E19, the expression levels of all ten *Slc22* genes have almost reached adult levels. To the exception of *Slc22a12*, all Log_2_ FC_ad_ absolute values at E19 were equal or lower than 2. Log_2_ FC_ad_ values calculated at E15 were much more variable, ranging between −7.16 for *Slc22a8*, reflecting a 143-fold enrichment in adult, and 3.79 for *Slc22a6*, corresponding to a 13.8-fold enrichment in E15.

Our findings are in accordance with a previous study showing high constitutive mRNA levels for *Slc22a5* and *Slc22a8* in adult rat choroid plexus in comparison to other *Slc22* transporters [41]. The Slc22a8 protein has been located at the apical membrane of the choroidal epithelium in rat [19,56]. Slc22a6 displayed the same cellular distribution when expressed as a fluorescent protein in adult rat choroid plexus tissue explants [19,56]. Inhibition experiments in choroidal cell monolayers pointed to apical Slc22 carriers, likely Slc22a8, as mediating the active efflux of antiviral nucleoside analogs across the blood-CSF barrier [57]. Slc22a6 and Slc22a8 transporters share a large number of exogenous substrates [58]. Our present data on their respective inverse profile of expression during development suggest that they sequentially contribute to organic anion clearance from the CSF. The switch from 22a6 to 22a8 in the embryo may reflect a need to adapt to changes in endogenous substrates at that period.

The expression of *Slc22a5* in choroid plexus was high and steady in the developing brain. This organic cation transporter will mediate toxic efflux from the CSF if the site of cellular uptake is apical. Its membrane localization is presently unknown. Because Slc22a5 transports carnitine with the highest affinity among Slc22 proteins, it could participate in the control of carnitine homeostasis in the developing brain. Although carnitine is a necessary cofactor of mitochondrial β-oxidation that generates energy from fatty acids, this function is not essential for the developing brain, because peripheral ketone bodies, rather than fatty acids themselves, fuel the brain in suckling animals [59]. Carnitine may function as an antioxidant [60] or a promoting and modulatory agent for synaptic neurotransmission, notably cholinergic transmission [61]. The high and steady expression of *Slc22a17* throughout development is noteworthy. This carrier is a cell surface receptor for the siderophore-binding protein 24p3, also known as lipocalin-2. In the brain the highest *Slc22a17* transcript levels are found in choroid plexus [62]. Murine Slc22a17 protein, when expressed in HeLa cells, binds both the iron-free and Fe-containing 24p3. It enables their internalization by endocytosis and leads to either apoptosis or increase in intracellular iron-content. Through its binding to 24p3, Slc22a17 may influence infectious/inflammatory processes at the blood-CSF barrier. As a siderophore-binding protein, 24p3 has potential bacteriostatic properties. It also forms a complex with the matrix metalloproteinase MMP-9 [63]. Both 24p3 and MMP9 are highly up-regulated in choroid plexus under inflammatory stimuli [64,65]. The relationship between Slc22a17, 24p3 and MMP9 at the choroid plexus, as well as the relevance of the high *Slc22a17* expression during development needs further investigations.

#### Slc15, Slc28 and Slc29 transporters

The Slc15 subfamily comprises proton-coupled oligopeptide transporters [66]. Among these, Slc15a2 transports peptidomimetic drugs such as the antiviral agent aciclovir or the β-lactam antibiotic cefadroxil. Slc15a2 protein was shown by immunocytochemistry to distribute at the apical and subapical domain of rat choroid plexus epithelial cells in culture [67]. Accordingly, studies using knock-out mice indicated that this transporter removes peptide substrates from CSF into the choroidal tissue [66]. The present analysis showed high levels of expression for *Slc15a2* that remained relatively unchanged during development (Figure 3D). These data support a protective role for this transporter in the developing brain toward neuropeptides in excess or peptidomimetic drugs. Slc15a3 and Slc15a4, which transport free histidine and endogenous di- or tripeptides, followed a developmental expression pattern similar to *Slc15a2* (data not shown). Their membrane localization and functions are currently unknown.

Slc28 and Slc29 transporters play critical roles in cellular uptake and release of nucleosides that are precursors for nucleotide biosynthesis. Even though brain penetration of nucleoside reverse transcriptase inhibitors used for AIDS treatment is not mediated by choroidal nucleoside transporters [57], these carriers can transport other anti-viral drugs and the anti-cancer drug gemcitabin [40,68]. Two concentrative Na^+^-coupled nucleoside transporters *Slc28a2* and *Slc28a3*, and four equilibrative nucleoside transporters, *Slc29a1 to Slc29a4* were identified in choroid plexus of E15 rats (Figure 3D). Most of these nucleoside transporters were expressed at levels close to adult levels from E15 onwards. Only *Slc28a3* and *Slc29a4* had lower transcript levels at E15 (p < 0.001). Functional transport and inhibition studies indicate a polarized localization of concentrative nucleoside transporters on the apical membrane, and equilibrative transporters on both the apical and basolateral membrane domains [69]. This subcellular distribution would imply that the concentrative transporters, possibly in conjunction with basolateral equilibrative transporters, fulfill neuroprotective functions by effluxing substrates from CSF rather than contributing to nucleoside entry into brain. This protective mechanism should be efficient already in the developing brain.

### Developmental profile of drug-metabolizing and antioxidant enzymes

Drug metabolizing enzymes include functionalization (phase I) enzymes that add or transform a functional group on lipophilic compounds. Resulting metabolites are usually less active and more polar. Cytochrome P450s (Cyp), in particular members of the Cyp1-3 families, flavin-containing monooxygenases (Fmo), and epoxide hydrolases play an important role in phase I of drug metabolism, by inactivating exogenous compounds including various drugs, pesticides, dietary-derived compounds and carcinogenic molecules. Drug metabolizing enzymes also include conjugation (phase II) enzymes that add a hydrophilic moiety such as glucuronic acid, sulfate, or glutathione to the parent drug or the phase I metabolite. Drug metabolizing enzymes of both phases have been associated with blood–brain interfaces [21,70].

#### Phase I drug metabolizing enzymes

**Figure 4:**
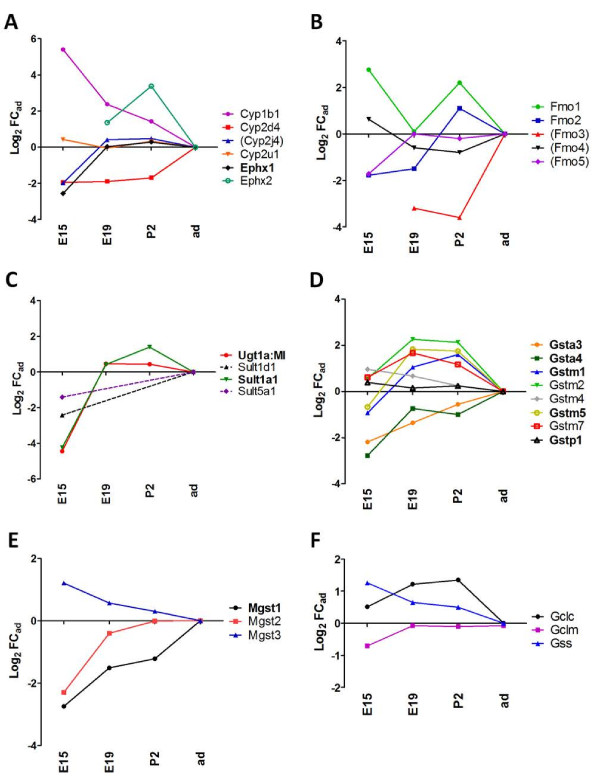
**Developmental profiles of drug metabolizing enzymes. (A)** shows gene expression profiles for different cytochrome P450 enzymes and epoxide hydrolases in the lateral ventricle choroid plexus. *Cyp2u1* and *Ephx1* display similar Log_2_ FC_ad_ during development, thus the developmental profile overlap on the graph. No expression for *Cyp2u1* was detected at E15. **(B)** shows gene expression profiles for flavin-dependent monoxygenase. *Fmo3* was undetectable at E15. **(C)** shows gene expression profiles for UDP glucuronosyltransferases and sulfotransferases. Ugt2a1 was detected in adult, but not at E15. **(D)** shows gene expression profiles for soluble glutathione-S-transferases, and **(E)** for microsomal glutathione-*S*-transferases. **(F)** shows expression profiles for genes involved in GSH synthesis and metabolism. MI, multiple isoforms. Other abbreviations, data analysis, and data expression as in Figures 2 and 3.

Among the more than 88 Cyps genes identified so far in the rat species (http://drnelson.uthsc.edu/rat.master.table.html), only four of them were detected in the lateral ventricle choroid plexus (Figure 4A). *Cyp2d4* transcripts were enriched at the adult stage, while *Cyp1b1* transcripts were enriched in embryos and perinatal stages. *Cyp2j4* and *Cyp2u1* expression did not change throughout development. Expression of microsomal epoxide hydrolase 1 (*Ephx1*) was high and steady in choroid plexus from E19 onwards, and only moderately lower (6-fold, p < 0.001) at E15. *Ephx2* transcripts, coding for the soluble form of epoxide hydrolase, were more abundant in choroid plexus from developing animals compared to adults (Figure 4A). All Fmo genes *(Fmo1* to −*5)* were expressed in choroid plexus from developing animals at levels close to adult levels, except for *Fmo3*, which was lower at P2 and E19 and undetectable at E15 (Figure 4B).

The present data suggest that Cyps play a marginal role in detoxification at the choroid plexus. This is in line with the low Cyp activities reported previously for choroidal tissue [70], and with the lack of Cyp1a1 activity in choroid plexus, the protein being detected only after inductive treatment and only in choroidal vessels [71,72]. Of note, Cyp1b1, known as the main Cyp isoform expressed at the blood–brain barrier in both rat and human [73,74], may have a specific detoxification function at the choroid plexus in early development, as its expression is highest in the pre- and postnatal period.

In contrast to Cyps, both Fmos and epoxide hydrolases appear to play significant detoxification roles at the blood-CSF barrier. The high level of expression of *Ephx1* in choroid plexus is in line with the high mEphx1 activity measured in the rat tissue [70] and with the strong immunoreactivity of the mouse choroidal epithelium [75]. *Ephx1* is well expressed throughout development, pointing to an efficient enzymatic protection towards carcinogenic epoxides and epoxide drug-intermediates from the embryonic period onwards. Ephx2 more specifically metabolizes lipid-derived epoxides, such as epoxyeicosatrienoic acids. Its age-dependent expression profile suggests a function in modulating lipid signaling during development.

*Fmo1* and *Fmo3*, whose transcripts were both detected in choroid plexus, are considered as the most important members in the Fmo family with regard to detoxication of foreign compounds [76]. *Fmo1* mRNA was previously detected in mouse choroid plexus by *in situ* hybridization [76]. The developmental profiles we observed for *Fmo* genes suggest that Fmo1-dependent biotransformation reactions support important choroidal detoxification pathways in the developing brain, which have never been explored. The significance of *Fmo2* transcripts encoding a truncated non-functional protein [76], and of *Fmo4* and *Fmo5* which were expressed at continuous but low level during development remains elusive in the context of detoxification.

#### Phase II drug metabolizing enzymes

Phase II of drug metabolism is catalyzed mainly by UDP-glucuronosyltransferases (Ugt), sulfotransferases (Sult), and glutathione-*S*-transferases (Gst). Similar to efflux transporters, the three enzyme families are multigenic families, composed of multiple isoforms with differential substrate specificities. This explains the broad substrate specificity of the overall detoxification process [21].

Among detoxifying Ugts, the highly homologous genes of the Ugt1a subfamily could not be distinguished by the microarray probes. Transcripts of these genes were highly and steadily present from E19 to adult (Figure 4C). Their level of expression was lower at E15. This is in agreement with enzymatic data showing that planar molecules, which are typical substrates of Ugt1a enzymes, are efficiently conjugated by choroid plexus epithelial cells both in adult and newborn rat [22,70]. The Ugt1a1 isoform plays an important physiological role in the hepatic clearance of bilirubin in addition to xenobiotics. Conjugation of bilirubin at the blood-CSF barrier could represent an efficient mechanism to prevent the cerebral accumulation of bilirubin in hyperbilirubinemia [77].

*Sult1a1* displayed a similar profile of expression, its transcripts being enriched from E19 to adult, compared to E15. In line with this apparent high level of choroidal expression, Sult1a1-dependent enzymatic activity measured in various human brain regions from fetuses of 15–20 weeks of gestation was the highest in choroid plexus [27]. Sult1a1 is also responsible for the inactivation of thyroid hormones. In addition to xenobiotic conjugation, choroidal Sult1a1 could regulate the levels of these hormones critical for brain development.

Gsts are homo- or heterodimeric enzymes divided into three families, the cytosolic, microsomal and mitochondrial transferases. Within cytosolic Gsts, the alpha (Gsta), mu (Gstm), and pi (Gstp) classes are mainly involved in drug metabolism and detoxification pathways [30]. Some microsomal Gsts (Mgst) were also examined in this study. Although their best known functions are related to the biotransformation of arachidonic acid metabolites, these isoforms are also active in detoxification [30,78]. Five out of seven detoxifying isoforms, *Gsta3*, *Gsta4*, *Gstm1*, *Gstm5*, and *Gstp1*, were highly expressed at the choroid plexus (Figure 4D). All mu isoforms, as well as *Gstp1* were expressed in choroid plexus of developing animals at similar or higher levels compared to adult. By contrast, transcripts of alpha *Gsts* were enriched in the adult (5- and 7-fold for *Gsta3* and *Gsta4* respectively) compared to E15. *Mgst1*, highly expressed in choroid plexus, showed a clear developmental up-regulation from E15 to adult, in contrast to *Mgst3* whose mRNA level was highest at E15 (Figure 4E). The diversity of *Gst* genes that we found expressed in choroid plexus is in accordance with the previous immunodetection of all alpha, mu and pi Gst classes in the adult rat choroidal epithelium [79-81]. The overall high levels of *Gst* transcripts observed during the perinatal period is in line with earlier studies showing Gst-positive cells in rat choroid plexus at embryonic day 16 [82] and with the presence of Gst pi in human fetal choroid plexus [83]. This transcriptional regulation parallels our previous enzymatic data showing that in both rat and human the choroidal conjugation of GSH with 1-chloro-2,4-dinitrobenzene, a substrate for multiple GSTs, was higher in fetus and newborn than in adult [22,23].

The rate of glutahione synthesis is in part determined by the activity of glutamate-cysteine ligase, which is composed of a catalytic subunit (Gclc) and a modulatory subunit (Gclm). These two genes, as well as the glutathione synthase gene (*Gss*) were expressed in choroid plexus from E15 onwards (Figure 4F). Transcripts levels in perinatal stages were equal or slightly higher to those in adult. These profiles suggest that the large capacity of the choroidal tissue to synthesize glutathione described in adults [84] is already acquired in embryos*.*

Both Mgst1, previously localized in epithelial cells of rat choroid plexuses [85], and Mgst3 have been described to have a peroxidase activity in addition to their glutathione-*S*-transferase activity [30]. *In vitro* experiments performed in MCF-7 breast carcinoma cells suggested that Mgst1 protects the cells against damages induced by cytostatic drugs or against SiO2 nanoparticle-induced oxidative stress and cytotoxicity [86]. Such antioxidant protection would be provided at the choroid plexus during development, by Mgst3 in earlier stages, then by Mgst1. These enzymes could reinforce the overall large antioxidant capacity of choroid plexus, which relies on various other enzymes present in this tissue (see below).

#### Antioxidant enzymes

Cells forming the blood-CSF barrier are exposed to oxidative stress of various origins. First, they face blood-borne pro-oxidant compounds. They also have an overall high mitochondrial activity, which is a substantial local source of oxygen-derived species. In addition, these cells are exposed to the reactive and potentially deleterious intermediate metabolites that can form as a drawback of phase I oxidative and reductive metabolism and of selected phase II reactions. Choroidal cells possess an extensive enzymatic machinery to inactivate all these reactive molecules. It includes superoxide dismutases (Sod), which inactivate superoxide anions, catalase (Cat), which cleaves hydrogen peroxide, and glutathione peroxidases (Gpx), which metabolize a large spectrum of peroxide species. Some isoforms of the latter enzymes require glutathione in their catalytic cycle, and thus function in conjunction with glutathione reductase (Gsr), which regenerates the reduced form of the thiol-containing molecule (reviewed in [87]).

**Figure 5:**
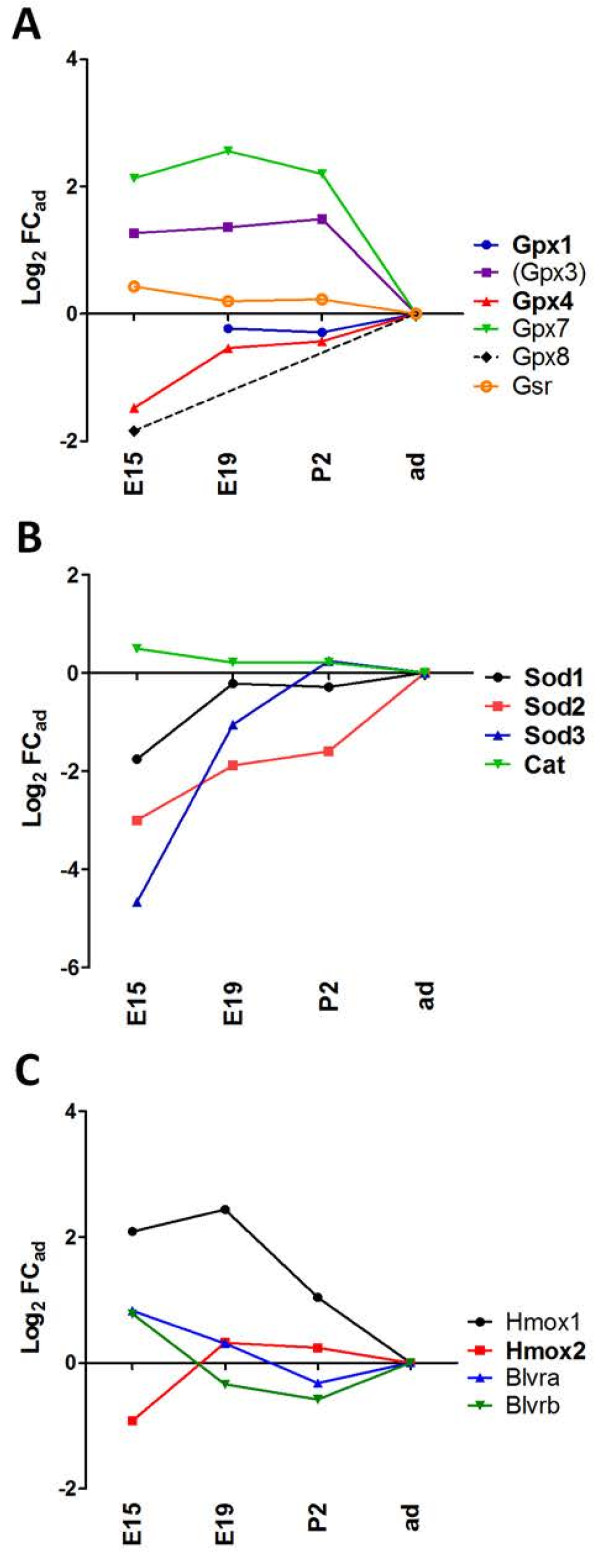
**Developmental profiles of other antioxidant enzymes. (A)** shows gene expression profiles for different glutathione peroxidases and glutathione reductase, **(B)** shows gene expression profiles for superoxide dismutases and for catalase, and **(C)** for heme oxygenases and biliverdin reductases. Abbreviations, data analysis, and data expression as in Figures 2 and 3.

Five out of eight Gpx genes identified in mammals (*Gpx1, -*3, -4, -7, and −8) and *Gsr* were detected in choroid plexuses (Figure 5A). All were expressed at similar or higher levels during the perinatal period compared to adult. At E15, Log_2_Fc_ad_ absolute values were lower than 2, indicating only limited variation at this early embryonic stage. *Gpx1* and *Gpx4* were expressed at high levels at all stages. Gpx1 is mainly devoted to the metabolism of hydrogen peroxide when produced in excess, as occurring in infections, while Gpx4 specifically inactivates membrane-integrated hydroperoxy lipids. It thereby counteracts the activity of lipoxygenases, and can modulate inflammatory responses [87]. Very high Gpx activities have been measured in rat choroid plexuses isolated from adults [88]. It is expected from our data that these enzymes are already functional at earlier times and therefore contribute to protect the developing brain against oxidative and inflammatory insults. The soluble Cu/Zn superoxide dismutase genes *Sod*1 and *Sod3,* as well as the *cat* gene were also highly expressed in choroid plexus both during the perinatal period and in adult (Figure 5B). Variations in expression of *Sod* genes were more heterogeneous in choroid plexuses in E15 animals than in subsequent developmental stages. A strong immunoreactivity for catalase was previously detected in rat choroid plexus as early as at E14.5 [89].

Mammalian cells display several other means of protection against reactive species, including the heme oxygenase (Hmox) pathway. Hmox proteins catalyse the degradation of heme, resulting in the formation of biliverdin, which in turn can be converted via biliverdin reductases (Bvr) into bilirubin. Intracellular unconjugated bilirubin acts as antioxidant, and the biliverdin/bilirubin redox cycle is considered as an important constituent of the cellular antioxidant defense system acting mainly towards lipophilic reactive species [77]. Moderate hyperbilirubinemia was found neuroprotective against focal ischemia to which neonates are especially susceptible [90]. Both stress-inducible *Hmox1* and constitutive *Hmox2* genes, as well as the *Bvra* and *Bvrb* genes were expressed in choroid plexus (Figure 5C). For all four genes, transcripts were detected at similar or slightly higher levels during development than in adults (log_2_ FC_ad_ values between −0.92 to 2.44). In line with our data, immunoreactivity of Hmox-2 usually considered as a neuronal isoform, has been reported in choroid plexuses of adult rats [91]. The steady expression of *Hmox2* and the concurrent enriched levels of *Hmox1* transcripts in embryonic stages represent a potent heme catabolic system able to generate biliverdin, not only in normal developmental conditions, but also in response to perinatal insults. In contrast to the liver, in which *bvra* and *bvrb* showed opposite age-dependent profiles of expression [92], both genes were expressed at similar levels in choroid plexuses from E19 to the adult stage. Thus the bilirubin/biliverdin antioxidant redox cycle is likely to be efficient in choroidal cells throughout development.

#### Transcription factors involved in the regulation of detoxifying genes

**Figure 6:**
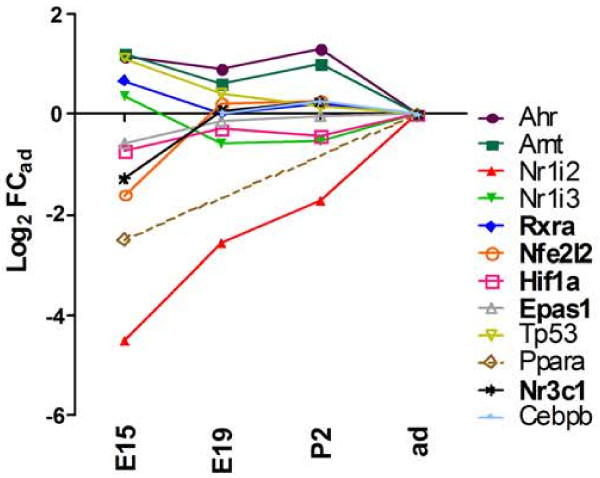
**Developmental profiles of transcription factors involved in regulation of detoxifying genes.** Gene expression profiles for transcription factors that regulate drug-metabolizing enzymes and efflux transporters, and for their co-regulators are shown. Abbreviations, data analysis, and data expression as in Figure 2.

Ligand and non-ligand activated transcription factors involved in the coordinated regulation of drug-metabolizing enzymes and transporters were incorporated in our study. They include the aryl hydrocarbon receptor (Ahr), the pregnane X receptor (*Nr1i2*), the constitutive androstane/activated receptor (*Nr1i3*), the peroxisome proliferator-activated receptor α (*Ppara*), the glucocorticoid receptor (*Nr3c1*), and the CCAAT/enhancer binding protein beta (*Cebpb*) reviewed in [29], and [30]. Ahr dimerizes with the Ahr nuclear translocator (Arnt, also known as Hif1b). Nr1i2, Nr1i3 and Ppara heterodimerize with the retinoid X receptor α (*Rxra*). The tumor protein p53 (Tp53) was added to the study as it was shown to directly mediate the transcriptional induction of *Abcb1* induced by genotoxic compounds [93]. The oxidative stress sensor nuclear factor erythroid 2-related factor 2 (*Nfe2l2*) and the hypoxia-inducible factor 1 alpha (*Hif1a*), all transcription factors involved in inductive processes mediated by redox imbalance and whose activation leads to the induction of detoxifying genes, are also reported. Four of these genes, *Rxra*, *Nfe2l2*, *Hif1a*, and *Nr3c1* were expressed at high levels in choroid plexuses. Apart from *Nr1i2*, whose expression was clearly upregulated from E15 to adult stage (p < 0.001), the choroidal expression of all transcription factors was remarkably steady throughout development (Figure 6). Absolute log_2_ FC_ad_ values determined at E19 were between 0 and 0.9.

Induction of choroidal drug metabolizing enzymes in choroid plexuses has been demonstrated only in adult rats, following treatment with polycyclic aromatic hydrocarbons, indicating that the AhR/Arnt-dependent pathway is functional at the blood-CSF barrier [72,94]. If functionality proves true for all factors throughout development, the panel of transcription factors that are expressed in choroid plexus suggests that following exposure to a large range of chemical xenobiotics the activity of drug metabolizing enzymes and transporters in the blood-CSF barrier can be induced in adult as well as in developing animals. Of note, the lower choroidal *Nr1i2* expression at E15 could be compensated by *Nr1i3* transcripts, as these two transcription factors regulate a number of common genes well expressed at the choroid plexus, such as *Ugt1a*, *Abcc* and cytosolic *Gsts*[95]. Similarly, the steady expression of *Nfe2l2*, *Hif1a*, and *Arnt* suggests that the neuroprotective functions of the blood-CSF barrier can be modulated in response to hypoxia and oxidative stress, whether occurring at early developmental age or in adult.

### Developmental profile of enzymes forming a barrier to neurotransmitters

**Figure 7:**
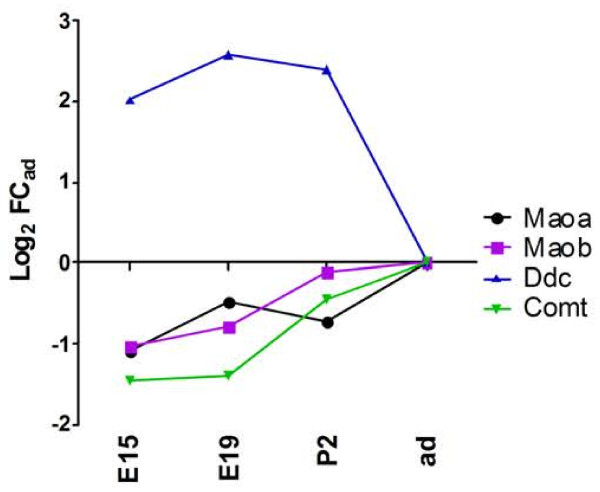
**Developmental profiles of enzymes forming a barrier to neurotransmitters.** Gene expression profiles for monoamine oxidases, dopa decarboxylase and catechol-O-methyltransferase are shown. Abbreviations, data analysis, and data expression as in Figure 2.

A function of enzymatic barrier to monoamine neurotransmitters, that involves three enzymes, has been ascribed to the choroidal epithelium [96]. The Dopa-decarboxylase (Ddc) catalyzes the decarboxylation of L-3,4-dihydroxyphenylalanine (L-Dopa) to dopamine, L-5-hydroxytryptophan to serotonin, and L-tryptophan to tryptamine. Monoamine oxidases (*Mao*) in turn metabolize and degrade the monoamine neurotransmitters dopamine, serotonin, and adrenalin. Catechol-O-methyltransferase (*Comt*) catalyzes the O-methylation of catecholamine neurotransmitters and catechol hormones, leading to their inactivation. It also shortens the biological half-lives of certain neuroactive drugs, like L-Dopa, alpha-methyl Dopa, and isoproterenol [97]. Choroidal *Ddc* expression was 4- to 5.5-fold higher in the choroid plexus of developing animals as compared to adult, while levels of *Maoa*, *Maob*, and *Comt* transcripts varied very little between E15 and adult (Figure 7). These data suggest that a choroidal enzymatic barrier towards neurotransmitters is already established before birth. If functional, as observed in adult [96], it could represent a protective mechanism preventing blood-borne monoamines from interfering with central neurotransmission.

### General comments

The combination of Affymetrix microarrays, RNA-Seq and qRT-PCR has enabled a comprehensive analysis of expression of choroid plexus genes involved in neuroprotection from embryonic through postnatal development compared with the adult. By their nature, microarrays are not comprehensive, but the additional use of RNA-Seq and qRT-PCR allowed the detection and quantification of genes not represented in the microarray. On the other hand some genes detected in the Affymetrix arrays were below or close to threshold in RNA-Seq. The quantitative estimates of changes in expression level of individual genes are more secure, because of the use of three independent methods.

The choroidal tissue microdissected from brain is predominantly constituted by the choroidal epithelial layer that forms the blood-CSF barrier, it also contains in smaller numbers fibroblasts and some myeloid cells forming the stromal core of the choroid plexus, and endothelial cells forming the choroidal vessels. Our study does not discriminate between these different cell types. However, apart from Cldn5 and Cyp1a1 which are localized in choroidal vessels [15,71], all other neuroprotective gene products for which immunohistochemical or functional analyses have been performed at the level of the choroid plexus have been identified in the choroidal epithelium. These include Cldn1, Cldn2, Cldn3, Cldn9, Cldn19, Abcc1, Abcc4, Slco1a4, Slco1a5, Slc22a6, Slc22a8, Slc15a2, concentrative and equilibrative nucleoside transporters, Ephx1, Ugt1a, several cytosolic and membrane-bound Gsts, cat, and enzymes bearing a barrier function to neurotransmitters [17-19,22,23,25,46,56,57,67],[69,75,80,85,89,96]. The present gene expression analysis therefore supports the concept that the blood-CSF barrier possesses an efficient neuroprotective machinery during the pre- and postnatal stages of development. This is reflected by the overall limited variation in the choroidal expression of efflux transporters and metabolizing/antioxidant enzymes between E19, P2 and adult animals (Table 2). Only a limited number of genes displayed a large age-dependent up or down regulation within this timeframe. The 16-fold higher transcript levels for *Abcc9 and Slco2a1* detected at E19 compared to adult likely reflect differences between the perinatal and adult functions of the blood-CSF barrier. These specificities remain to be investigated. The variation in gene expression relative to adult levels is more pronounced at E15 than at perinatal stages of development (Table 2). Thirty percent of the neuroprotective genes analyzed in the present work were expressed at levels at least 4-fold lower at E15 compared to E19, reflecting some degree of maturation of the functions associated with these genes between early embryonic and late fetal stages. By contrast, transcript levels were 4-fold higher or more in E15 compared to E19 for only ten percent of the genes. Given the growing evidence for the important role of choroid plexus in early stages of brain development [4,98] these particular choroidal transporters and enzymes may be involved in brain development processes rather than in neuroprotection *per se*.

**Table 2:** Average amplitude of fold changes in neuroprotective gene expression levels in E15, E19 and P2 animals relative to adults in the choroid plexus

	**TJ proteins**	**Transporters**	**Enzymes**
**E15**	5.9 ± 1.4	23.5 ± 9.2	5.1 ± 1.1
**E19**	2.5 ± 0.6	3.6 ± 0.9	2.1 ± 0.2
**P2**	1.9 ± 0.3	3.2 ± 0.7	1.9 ± 0.2
**E15 vs. E19**	*	*	**
**E15 vs. P2**	**	**	***
**E19 vs. P2**	ns	ns	ns

Data are expressed as mean ± SEM of trend-independent FC calculated back from Log_2_FC_ad_ “absolute” values for all the genes (but three), analyzed in this study and illustrated in Figures 2, 3, 4, 5, 7. *Slc22a12, Fmo3 and Ugt2a1* were excluded because FC_ad_ values at E15 could not be calculated (see figure legends). “Absolute” values of Log_2_FC_ad_ correspond to the value reported in the different figures, except that negative signs were removed. This table thus illustrates the FC amplitude independently of the variation trend, whether expression levels are higher or lower in earlier stages compared to the adult.

*, **, ***Statistical differences between developmental stages, analyzed by Kruskal-Wallis followed by Dunns multiple comparison test, *p <* 0.05, 0.01, 0.001, respectively. ns: not statistically different.

## Conclusions

The present data provide an overview of the multiple enzymatic and transport systems expressed in choroid plexus, which in conjunction with the epithelial tight junctional complexes, contribute to the protective barrier functions of the blood-CSF interface. However, it remains to be confirmed by *in vivo* testing with drugs and other xenobiotics if these systems are as functionally efficient in the developing brain as in the adult. Further characterization of these proteins to determine their localization and efficiency during development will enable more reliable risk assessment for drugs used to treat perinatal diseases. Evidence for the choroidal expression of various transcription factors involved in the induction of neuroprotective genes opens new pharmacological perspectives to improve neuroprotection at the interfaces between the blood and the central nervous system in the context of both perinatal injuries and environmental toxicity.

## Endnotes

^a^ Gene official symbols (in italic), as defined on the NCBI website, other common gene or protein names, and accession numbers (NM; number mRNA) for all genes analyzed in this study are listed in Table 1. Symbols referring to human genes or proteins are written in capital letters.

## Abbreviations

ABC: ATP-binding cassette; CSF: Cerebrospinal fluid; FC: Fold changes; qRT-PCR: Quantitative real-time polymerase chain reaction; Slc: Solute carrier; TJ: Tight junctions.

## Competing interests

The authors declare that they have no competing interests.

## Authors’ contributions

IK and NRS carried out the Affymetrix and qRT-PCR analyses. SAL and KS carried out the Illumina analysis. IK, SAL and JFGE performed the statistical analysis. NS, NRS and JFGE conceived and designed the study. IK and JFGE drafted the manuscript. All authors read and approved the final manuscript.

## Supplementary Material

Additional file 1 List of primers, amplicon sizes and MgCl2-concentrations used for qRT-PCR. Click here for file
