# Supplementary material for: Developmental changes in the transcriptome of the rat choroid plexus in relation to neuroprotection
Source: Fluids Barriers CNS. 2013 Aug 1;10:25. doi: 10.1186/2045-8118-10-25 (PMC3737068; doi:10.1186/2045-8118-10-25)
Supplement: Additional file 1 — List of primers, amplicon sizes and MgCl2-concentrations used for qRT-PCR. [file 2045-8118-10-25-S1.pdf]

Additional file 1(PDF)

List of primers, amplicon sizes and $MgCl_2$-concentrations used for qRTPCR

**Gene name**	**Forward primer 5'→ 3'**	**Reverse primer 5'→ 3'**	**size (bp)**	**$MgCl_2$**
AraB (*E. coli*)	atcccctgatcggtaaagca	acgcctgaaaggggtgatta	126	4 mM
Cldn1	gctaagctgctaaccctgtgg t	tctggcaagtctagcagtttgtg	202	3 mM
Cldn 3	actaccaaccgtcgatgtaccc	ccattcgacttggacagttcct	188	3 mM
Cldn 5	tacatgctaacctgaaagggca	aggaggaaggcaacccttctaa	196	4 mM
Cldn11	tgaaggaactgaaccaagcaga	aaacagcactgcttcaagatcg	208	3mM
Cldn 12	tttaagtgttccagattgggca	aggttgtctgacacactggctc	181	4mM
Cldn 19	caagaccccgcccacacgtg	agcaccgtgcgctgagatcc	195	3mM
Occludin	gactgggtcagggaatatccacc	agcagcagccatgtactcttcac	192	3mM
Tjp1	ttcttgcaaagtatcccttctg	ccacaaaagaaatcctttcaca	165	4mM
Tjp2	ctgaatgcatgaggatcttgga	ccacaaagtcagaggcttgaga	188	4mM
Tjp3	gggaacagcacacggccacac	cggccctccaggtaccacgtc	122	4mM
Abcc4	ttccccttcgaccttatcct	taggcagctgttgtcagtgg	124	4mM
Abcg2	acccacaggcggaggcaagt	gcaggttgaggtgccccgttt	105	3mM
Sco1a4	ctcatattcagggggcttca	cggtggcaaacagttctttc	148	3mM
Slco1a5	ttctactgccctgtgcaaga	agcagcatgaaacgacacac	147	3mM
Slco1c1	tgtcttgctggctgtcaatc	atcatgcctgaccagtttcc	111	4mM
Slc22a17	aatgactctcacggggattg	gcagcaaggagggtactgag	138	3mM
Slc22a8	ggcccttaccggagactatc	tcttcagcgggattatttgg	108	4mM
Ephx1	gctgtgctctgaatgactct	ctctccaggcctccatcc	109	3mM
